# Supplementary material for: FGFR2 overexpression and compromised survival in diffuse-type gastric cancer in a large central European cohort
Source: PLoS One. 2022 Feb 15;17(2):e0264011. doi: 10.1371/journal.pone.0264011 (PMC8846517; doi:10.1371/journal.pone.0264011)
Supplement: Attachment — Submitted filename: Response to Reviewers.docx [file pone.0264011.s005.docx]

To

Editor

*PlosOne*

Kiel, 14.01.2022

**Submission of a manuscript**

Dear Sir or Madam,

please find attached our revised manuscript, entitled “FGFR2 overexpression and compromised survival in diffuse-type gastric cancer in a large central European cohort”, which we are submitting to *PlosOne*.

We thank the editorial board for giving us the chance to revise our manuscript and also want to thank the reviewer for the critical comments. We have implemented changes to our manuscript based on the comments and believe this has improved the quality of our manuscript. All changes are highlighted in red font.

Editorial comment:

Please pay a careful attention to the critical comments of the Reviewer.

Response: All reviewer comments were considered. We also added four Supplemental Tables, including a Supplemental Table with the minimal data set (Table S1).

Specifically, discuss the results obtained more thoroughly in comparison with other, published studies including a large meta-analysis referred to as was the primary aim of the research.

Also, please consider and discuss the potential clinical implications of your main finding of the negative correlation of FGFR2 expression to patient outcome in diffuse gastric cancer.

Response: The discussion was amended, including an additional section on the clinical significance of our findings.

Also, could any correlation of FGFR2 status to clinical progression of gastric cancer patients be found? Did observed lymphatic invasion correlate with the metastatic pattern of diffuse gastric cancer?

Response: As shown in Table 1, T category and UICC tumor stage as surrogates for tumor progression, did not correlate with the FGFR status. We also carried out additional statistics. However, no correlation was found between pL- and pM category in diffuse type GC (Table S3).

Mechanistically, the observed heterogeneity of FGFR2 staining could be discussed in more detailed way.

Response: We have added a section on tumor heterogeneity in the discusson. We were able to demonstrate in our work signs of intratype and intraprimary heterogeneity at the genetic level as well as that in protein expression of FGFR2.

Did cellular compartmentalisation (membrane staining vs intracellular staining analysed in the study) of the FGFR2 IHC correlate with any characteristics of diffuse gastric cancer compared to intestinal type.

Response: We performed additional statics and did not find any corelation between intracellular localization of FGFR2 immunostaining any clinicopathological patient characteristic (Table S3). The data were added to the revised manuscript.

Additionally, the cohort from the Kiel area may not represent the whole European population although certainly to it.

Response: We added the following sentence to the discussion: “Our study has some limitations. First, the data were obtained only from a single center, which may limit the transferability to other white patient populations. A multicenter study is necessary to validate our data.”

The term "caucasian" may not be appropriate.

Response: We acknowledge the recent discussion on the terms “Caucasian” and “White". The terms “European” and “Caucasian” were replaced by “White” where appropriate.

Reviewer #1:

1) The study analyzes FGFR2 expression in gastric cancer using immunohistochemistry and chromogenic-in-situ hybridization techniques. It comes from the Department of Pathology at Christian-Albrechts-University in Kiel, Germany. The list of authors does not seem to include any surgeons, oncologists nor epidemiologists.

Response: The authors of our manuscript adhere to the criteria based on the International Committee of Medical Journal Editors, i.e., (1) substantial contributions to conception and design, acquisition of data, or analysis and interpretation of data; (2) drafting the article or revising it critically for important intellectual content; (3) final approval of the version to be published, and

(4) agreement to be accountable for all aspects of the work in ensuring that questions related to the accuracy or integrity of any part of the work are appropriately investigated and resolved. No surgeon, oncologist or epidemiologist fulfilled these criteria to justify co-authorship.

2) The FGFR2 expression was found to be very heterogenous. The FGFR2 protein expression did not correlate with patient survival in the entire cohort. After subanalysis, the authors found a correlation between FGFR2 expression and patient outcome in diffuse type gastric cancer. The subset of patients (FGFR2-positive and diffuse type) constitutes a small subset of patients, as the authors conclude. Further, the main finding of the study with Central European patient cohort really was that FGFR2 overexpression is associated with shorter survival in diffuse type of gastric cancer. The authors do not elaborate this further. So what? What are the clinical implication? Differences in Asia vs. Europe?

Response: We amended the discussion.

3) In the literature, there several reports on the correlation (or lack of it) between FGFR2 expression and patient survival. This study, unfortunately, does not clear the discrepancy between previous findings.

Response: As mentioned above in response to the editorial comments, the discussion was revised and an additional section on the clinical implications of our work was added. We have also attempted to provide a clearer comparison of the differences between our European and the previously studied Asian cohorts. The leading differences we see are the different distribution of tumor types and the better prognosis of the Asian patients.

4) In several parts of the manuscript, linguistic revision is needed.

Response: The manuscript was proof read by Whiley editing service. However, professional editing focused solely on linguistic errors and grammar and not on the scientific content.

Minor comments:

Line 81: The coverage and reliability of collecting follow-up data from interviews of general practitioners remain limited.

Response: We agree with the referee.

Figure 1 is lacking the bars.

Response: The bars were added to the figure.

Additional comments:

- During revision we noticed that we had used the 7^th^ edition of UICC, which is outdated. We therefore revised statistics using the 8^th^ edition of the TNM Classification of Malignant Tumors. The revision did not result in any significant changes.
- We also reworded the title to make it more concise.

We confirm that the publication is approved by all authors. The authors hereby state that the manuscript has not been published and that, if accepted, it will not be published elsewhere in the same form, in English or in any other language, including electronically without the written consent of the copyright-holder. All authors have contributed to the work described sufficiently to be named as authors. We hereby confirm that all presented material is original research. The authors declare no conflict of interest.

In looking forward to your response

Yours sincerely

Prof. Dr. med. C. Röcken
